# Internet of Things for beyond-the-laboratory prosthetics research

**DOI:** 10.1098/rsta.2021.0005

**Published:** 2022-07-25

**Authors:** Hancong Wu, Matthew Dyson, Kianoush Nazarpour

**Affiliations:** ^1^ Edinburgh Neuroprosthetics Laboratory, School of Informatics, The University of Edinburgh, Edinburgh EH8 9AB, UK; ^2^ Intelligent Sensing Laboratory, School of Engineering, Newcastle University, Newcastle upon Tyne NE1 7RU, UK

**Keywords:** myoelectric control, internet of things, abstract decoder, Naive Bayes classifier

## Abstract

Research on upper-limb prostheses is typically laboratory-based. Evidence indicates that research has not yet led to prostheses that meet user needs. Inefficient communication loops between users, clinicians and manufacturers limit the amount of quantitative and qualitative data that researchers can use in refining their innovations. This paper offers a first demonstration of an alternative paradigm by which remote, beyond-the-laboratory prosthesis research according to user needs is feasible. Specifically, the proposed Internet of Things setting allows remote data collection, real-time visualization and prosthesis reprogramming through Wi-Fi and a commercial cloud portal. Via a dashboard, the user can adjust the configuration of the device and append contextual information to the prosthetic data. We evaluated this demonstrator in real-time experiments with three able-bodied participants. Results promise the potential of contextual data collection and system update through the internet, which may provide real-life data for algorithm training and reduce the complexity of send-home trials.

This article is part of the theme issue ‘Advanced neurotechnologies: translating innovation for health and well-being’.

## 1. Introduction

The loss of the upper limb affects three million people all over the world [1,2]. People with upper-limb difference will face severe challenges in performing activities of daily living [3]. For centuries, prostheses have been used to restore the functions of the missing limb, e.g. cooking, feeding and dressing. Fully restoring the appearance and the function is challenging due to the large number of degrees of freedom involved [4]. Today, the design of multi-functional prostheses and natural prosthetic control algorithms still attract lots of interest and effort in laboratories.

Most prosthetic techniques are developed and tested within a controlled laboratory environment [5,6]. Upon device calibration, data collection and user training, most participants can control the prosthesis or the virtual test space reasonably well. However, previous studies indicate that myoelectric prosthesis control in a laboratory environment is not representative of real-life scenarios beyond the laboratory [7–11]. The variability in muscle activity and limb position, between many other factors, affects the generalization of control. As such, we need a new experimental paradigm by which the performance of the laboratory innovation can be tested beyond the laboratory.

In addition, in current clinical practice, once a user returns home after receiving a prosthesis, it is difficult, if not impossible, for the clinicians to monitor the prosthesis use remotely or to reconfigure the device if needed. This lack of oversight or maintenance degrades the comfort and functionality of the device over time, which increases the rate of prosthesis abandonment [12].

To bridge the gap between laboratory research, clinical practice, prosthesis use in home environment studies are designed to investigate prosthetic behaviours in real life. The experimenter sets up the prosthesis and sends it to the user’s home [13–15]. Results can be recorded through surveys [13,16–18] and standard clinical trials [13,16,18]. Further data, such as wear time and number of grips, may be recorded with embedded electronics [5,13,16,17]. Equipped with large hard disks, recent systems also allow the recording of raw myoelectric signals and video clips during home trials [15,19], which may provide more quantitative and qualitative information for further analysis. Similar to the clinical practice, current home trials may still rely on regular visits to the laboratories or clinics for user training and device maintenance. The user normally sends their feedback through email or by telephone call, which has limited bandwidth and the hysteresis effect [20]. Also, the user cannot accurately add contextual information to their prosthesis use data. Additionally, the experimenter may find it difficult, if not impossible, to provide technical support to the user remotely.

An Internet of Things (IoT) setting provides new possibilities in data exchange and information integration [21]. It facilitates the collection and transfer of data over a wireless network without human intervention. In recent years, IoT has been applied to the healthcare systems for remote health monitoring, such as heart rate, body temperature and blood pressure [22–24]. In terms of assistive technology, it has been used for remote data collection and security checks [25–27]. The success stories around IoT indicate a potential solution for home-use prosthetic studies to bridge the gap between the experimenter and the user. For example, Williams *et al.* [28] designed *Ubi-Sleeve* that enabled people wearing lower-limb prostheses and other stakeholders to record various signals, e.g. temperature, humidity and prosthesis slippage behaviour, from inside the socket regularly. Also, Fukuda *et al.* [29] showed the feasibility of transmitting myoelectric signals and sensor data up to a cloud server for determining a grasp pattern and receiving the motor command to actuate a robotic gripper. As such, parts of the prosthetics industry have embraced the change and introduced to the market connected solutions, e.g. Linx^TM ^by Blatchford.

Here, we demonstrate the feasibility of an IoT-enabled system for beyond-laboratory prosthetics research with the vision that the proposed conceptual framework can underpin the future of remote prosthetics care. The device is connected to a cloud server, which receives and synchronizes the data with the user feedback. We developed a dashboard and a control panel on the server for each prosthetic device, so that users can have visual feedback on their myoelectric signals and adjust the system, if required. The platform enables an experimenter to analyse the data and refine the control algorithm in a personalized way. We show the feasibility of remote collection of contextualized data as well as system reconfiguration.

## 2 Methods

**Figure 1.**
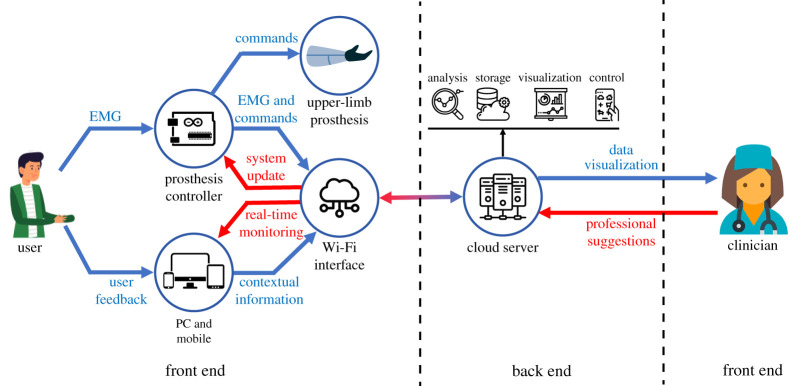
Schematic diagram of the Internet of Prostheses. (Online version in colour.)

The system developed in this study includes the upper limb prosthesis in an IoT architecture, as shown in figure 1. The prosthesis controller and the smart devices (PC and mobile) are with the user to control the upper-limb prosthesis and collect control signals and contextual information. They can work independently without internet connection, but connecting the device to the cloud server allows the device to upload the collected data and receive system updates through Wi-Fi communication. The cloud server is developed on the Thinger.io platform to allow remote data collection, storage and visualization as well as the control and update of devices. Users and clinicians can access to the server anywhere by logging into its web portal, so the experiments are no longer limited in the laboratory because of the hardware constraints. More details about each module in the schematic diagram are introduced in the following sections.

### (a) System description

#### (i) Hardware

**Figure 2.**
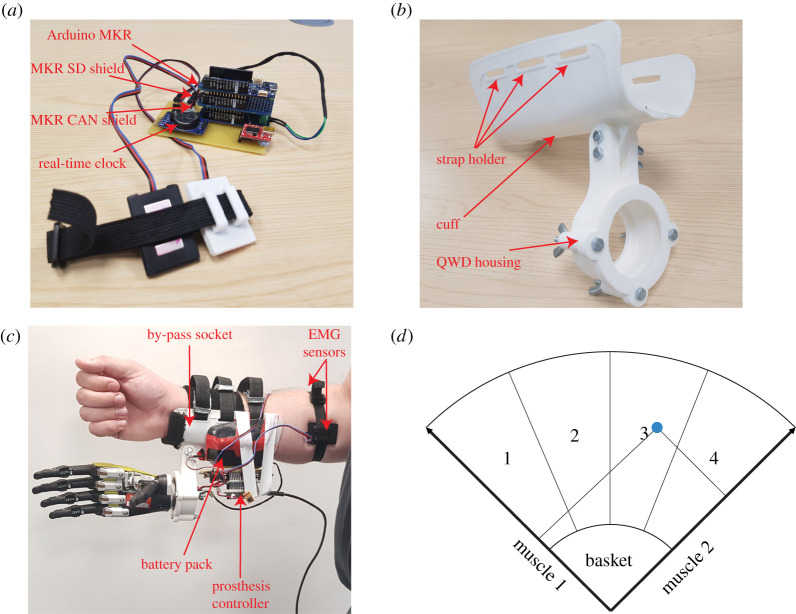
Overview of the internet-enabled prosthesis. (*a*) The prosthesis controller developed on the Arduino; (*b*) three-dimensionally printed by-pass socket; (*c*) portable upper-limb prosthesis for able-bodied participants; and (*d*) the myoelectric interface that maps the muscle activity to four grasps on the prosthesis. (Online version in colour.)

The prosthesis controller was implemented on an Arduino MKR WIFI 1010 (Arduino LLC, USA) because of its low-cost and accessibility [24]. This system enables two-channel myoelectric recording with a 500 Hz sampling rate, signal pre-processing with bandpass filters and notch filter, abstract decoding for real-time prosthesis control. For this work, we used the smoothed envelop of the EMG signal, which is of the order of a few Hz. As such, a lower sampling rate, violating the Nyquist frequency, does not contaminate the data by aliasing [30]. Besides, a wireless communication module was developed based on the Protoson (PSON) protocol [31], which enabled the bi-directional binary data transmission between the server and the device. Furthermore, a micro-SD shield (Arduino MKR SD Proto Shield) and a real-time clock module (HW-084) were connected to the system for data storage and synchronization. While the prosthesis was used, control data were logged to the micro-SD card with timestamps, so users could save the data onboard if Wi-Fi communication was not available. A prototype of the internet-enabled prosthesis controller is shown in figure 2*a*.

We three-dimensionally printed a by-pass socket (figure 2*b*). It included a cuff and a two-part quick wrist disconnect (QWD) housing. The cuff was printed flat and thermoformed to encapsulate the lower forearm using a heat gun. The print orientation of the cuff gave it slight intentional flexibility to allow the brace to fit all participants easily. Three adjustable straps were added to the top of the cuff. The cuff also features an attachment site for the QWD housing to hold the Robo-limb (Ossur, Iceland). The two housing parts were printed separately and affixed to the cuff with bolts. Batteries and the prosthesis controller were placed behind the QWD housing to reduce the length of the cables. An overview of the hardware is shown in figure 2*c*.

#### (ii) Prosthesis control algorithm

We could use any prosthesis control method to demonstrate this IoT-based setting. In this proof of principle study, for the first time, we implemented the abstract control method [32,33] for the control of a prosthesis in a real-time experiment. With the support of a computer-based myoelectric interface, users can learn novel ways to map their muscle activity to commands to actuate a prosthesis. We previously described the interface in [10] and [33]. For completeness, we briefly describe the controller here. Figure 2*d* shows a circular cursor on a two-dimensional task space. The position of the cursor is determined by the mean-absolute value (MAV) of the myoelectric signals from two muscles. Participants can move the cursor from the lower basket (rest state) to a target and hold the cursor within the target for a fixed dwell period to activate a preset grasp on the prosthesis. We assigned power, tripod/pinch and point grasps, and hand open to targets one to four, respectively. We set the state machine such that the prosthesis can accept a new grasp command only when the hand is in the open state and the cursor is in the rest basket. This way we removed the possibility of inadvertent grasps.

#### (iii) IoT cloud portal and server

The cloud server was developed using the Software as a Service (SaaS) model [34]. It was deployed on the Thinger.io platform [31] that handled data collection, data visualization and device reconfiguration. All registered stakeholders can access the server through a web browser. No local software installation was required. To satisfy the requirements of different stakeholders, the platform offers the possibility of stakeholder-specific dashboards at various granularity. Specifically, two web-based dashboards, one for a user and the other for an experimenter, were developed using the representational state transfer (REST) application programming interface and were saved as templates. We could include new members in the experiment by simply applying the template to their accounts or by personalizing it according to user preferences. The data were securely stored on an Amazon DynamoDB database and separated into virtual storage spaces called *Data Buckets*. For privacy and security reasons, data buckets were created and managed for each user individually. When a prosthesis controller was connected to the server, its control data were synchronized to the user’s data buckets automatically. Users could log into their own accounts to monitor and reset their prostheses, while the clinician could read, modify and download data from users they were responsible for.

### (b) Participants and preparation

Three able-bodied participants took part in this proof-of-principle study. They were free from any known neurological or motor disorders. They all had extensive experience of computer-based abstract myoelectric control. This study was their first experience of wearing a prosthesis by-pass socket and controlling a commercial myoelectric prosthesis.

Myoelectric sensors were placed on the skin, targeting the flexor carpi radialis (FCR) and extensor carpi radialis (ECR) muscles. Normalized mean-absolute values of the myoelectric signals were calculated and used as control signals following the method described in [33]. Before the experiment and as a familiarization block, participants wore the socket and the prosthesis and practised with the cursor control on the computer interface. The power supply of the prosthesis was off during this block. This way we ensured that the familiarization block is representative of the main study, in terms of the load on the arm.

The familiarization block comprised two stages. The first stage included a practice run and then 40 trials. First, participants practised the movement of the myoelectric cursor in the abstract decoding task space (figure 2*d*) for 2 min. After that, in each trial, a target was selected and highlighted on the interface, and participants moved the cursor from the basket to hit the selected target within 1.5 s. Participants were instructed to achieve as many trials as they could. They needed to achieve more than 70% trials for each target in order to clear the stage. Throughout this stage, they could see the cursor on the computer screen. At the end of the stage, participants had a 2 min or longer break to avoid fatigue. They were instructed to place their forearm on their legs or the handle of the experimental chair to rest their muscles. The second stage closely followed the first but with the difference that the cursor was no longer visible. The second stage mimicked the prosthesis control in a practical scenario.

### (c) Functional assessment

A real-time control *pick and place* experiment was carried out to investigate the functionality and robustness of the proposed system. The prosthesis controller was connected to the server and data were recorded. This study comprised 16 blocks, comprising two groups of eight. In each block, participants were required to grasp, lift and relocate a series of objects. They were also required to press the touchpad of the experimental laptop with a point grip to indicate the start and stop of the block. Only in one block, a participant did not click the touchpad properly. This block was removed from the analysis.

In the first eight blocks, the objects were a compact disc and a drink can. As such, the grasp order was point, tripod, power and point. The order in which they picked the disc and the can was decided by the participant. In the second eight blocks, the objects were a ball and the same drink can. The grasp order in the second eight block was point, pinch, power and point. Similarly, the order in which they picked the ball and the can was decided by the subjects. Note that the change from tripod to pinch grip on the prosthesis was carried out on the server remotely, during the experiment, demonstrating the flexibility of the proposed system. If participants lifted an object with a grasp that was not associated with the object, they had to submit user feedback to the server using the dashboard to report the grasp they intended to use. To avoid fatigue, due to bearing the weight of the hand, a 1 min break was included between each block, during which participants could put down their arms to rest the muscles. Participants could ask for a longer rest if they still required more rest.

### (d) Data analysis and system update based on real-life data

The data buckets were exported to the analysis laptop. For each participant, the myoelectric control signals were synchronized with the motor commands and the user feedback based on the timestamps. Motor commands without unexpected grasp labels were considered as the correct commands. Also, we evaluated the feasibility of updating the control algorithm based on real-life data. The correct motor commands in the pick and place experiment were used as the training data. Their angle v from cursor positions was extracted to train three 2-class Naive Bayes classifiers. The optimized decision boundaries between each two adjacent targets located at the position where the angle v has equal probability density between two targets. The new decision boundaries were verified by the unexpected motor commands.

## 3. Results

### (a) Online dashboards for device control and data visualization

The user interface, user feedback dashboard and clinician interface were developed for this study. Each prosthesis controller has an independent user interface and a user feedback dashboard for data visualization, device control and contextual data reporting.

**Figure 3.**
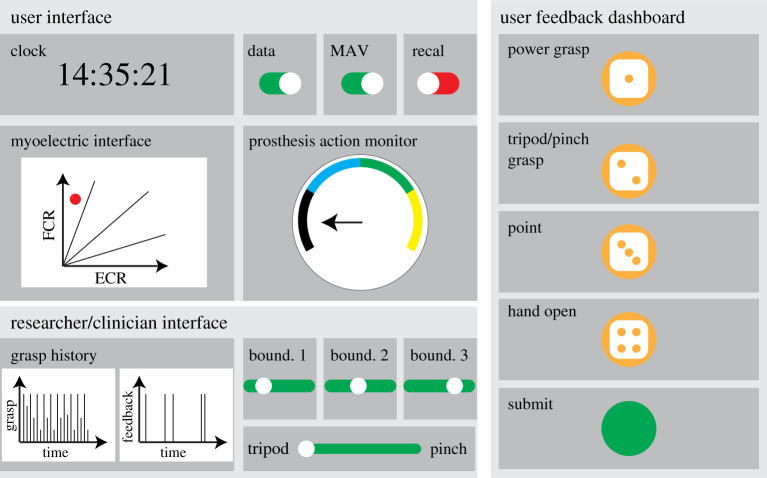
Overview of the interfaces. (*a*) User interface for real-time prosthesis monitoring and recalibration. (*b*) User feedback dashboard for contextual information collection. (*c*) Clinician interface for time-series data visualization and system reconfiguration. (Online version in colour.)

A user interface (figure 3*a*) includes a prosthesis monitor and an MCI, which shows the current status of the upper-limb prosthesis and the corresponding control signals in real time. It provides the visual feedback of the muscle contraction when the user is trying to control the prosthesis, so it may be used for in-home myoelectric training and the evaluation of the prosthesis settings, such as the calibration levels. The recalibration switch on the screen allows the user to recalibrate the corresponding device following our standard calibration protocol [10]. Regarding the ethical considerations, such as privacy, the user can stop sharing the data with the server by turning off the Data streaming and MAV streaming switches, which stops the device from recording control data and EMG signals but maintain its functions in prosthesis control.

A user feedback dashboard (figure 3*b*) allows the user to upload the contextual information when they are using the prosthesis. The user presses the four buttons on the top of the dashboard to report which grip they intend to select through the previous muscle contraction, and our system will label the selected grip to the corresponding MAV data. In this way, the user can provide labels for the practical muscle contractions during daily activities and we are able to collect the information remotely for user behaviour analysis and algorithm training. There is a questionnaire on the dashboard for the user to provide contextual information, such as where they are using the prosthesis. As the pick and place experiment was carried out in the laboratory, we did not require the participants to use this function during the tests.

The clinician interface (figure 3*c*) can be used to monitor and manage all prosthetic devices. It has time-series charts to show the motor commands, user feedback and corresponding timestamps from the data buckets for each device, so when the prostheses have been used and what activities they have been used for can be monitored remotely. The scale of the time-series charts is adjustable, so it can be used to analyse grasp selection for a specific task or to monitor the time of device usage in a longer term. The control panel next to the time-series charts can change the device settings for the selected participant, so the device reconfiguration can be carried out without physical access to the device.

### (b) Contextual data collection and synchronization

**Figure 4.**
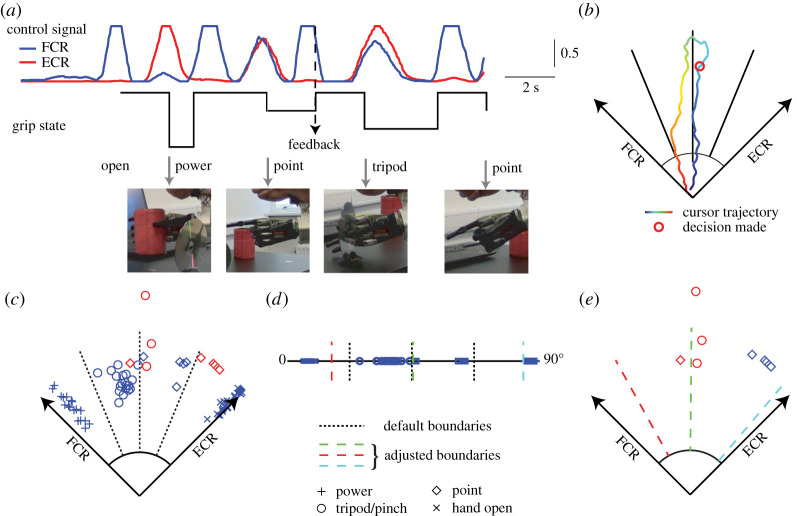
Example data for a pick and place trial. (*a*) The MAV signals and motor commands during a pick and place trial. (*b*) Labelling a motor command by the user feedback. An example of a grip decision and its corresponding cursor trajectory. (*c*) Scatter plot of the motor commands of one participant during the pick and place tests. The scatters show the contraction levels of two muscles when the participant hits the targets. Shape and colour of the scatters indicate the expected motor commands and if the hand movement meets the expectation, respectively. (*d*) One-dimensional angular map of the scatters used to train the naive Bayes decoders. (*e*) Customized decision boundaries for the participant. (Online version in colour.)

Data stored in the data bucket can be both visualized on the dashboards and downloaded for offline analysis in CSV, ARFF or JSON formats. Figure 4 shows an example of a pick and place trial operated by one of the participants. After clicking the keyboard with the point grip, the participant picked up the can and relocated it to the target label using the power grip. When picking up the disc, the prosthetic hand grasped with the point grip, which was not expected by the participant. They therefore submitted feedback to the server with their smartphone in the left hand, and then finished the rest of the trial following the instructions. With the timestamps, MAV signals, motor commands and user feedback can be synchronized, as in figure 4*a*. The user feedback was automatically labelled to its previous motor command as the grip that the participant intended to make. The corresponding cursor trace and the cursor position where the decision has been made (figure 4*b*) were saved as a new dataset so that prosthetic control data during practical activities can be labelled and collected remotely. Representatives for successful trials and also trials in which the participants provided feedback can be found in the electronic supplementary material, video.

### (c) Offline data analysis and system customization

Based on the experimental data, we trained the system to fit the participants’ short-term adaptation to the abstract myoelectric controller. The decision-making points for grasp commands (figure 4*c*) were decoded to its phase angle in the MCI (figure 4*d*). With the naive Bayes classifier, new decision boundaries were calculated for adjacent targets in the MCI. For the selected participant, four out of eight unexpected grasps were in the correct targets if we applied the customized decision boundaries (figure 4*e*). Participants may develop different strategies for abstract myoelectric control through adaptation or learning, so the customized decision boundaries for the three participants (table 1) may be different. The customized decision boundaries can be updated to the prosthesis controller through the clinician interface (figure 2*d*) remotely through the researcher interface. Table 2 shows the number of unexpected grip feedback that locate the correct target if customized Bayes decision boundaries are applied.

**Table 1.** Customized decision boundaries based on the contextual data for each participant (P).

	boundary 1–2	boundary 2–3	boundary 3–4
default	22.5°	45°	67.5°
P1	19.1°	43.2°	64.9°
P2	16°	45.4°	85.3°
P3	20.7°	45.8°	69.5°

**Table 2.** The number of user feedback and the number of corrections after adjustment of the decision boundaries.

	participant 1	participant 2	participant 3
no. of feedback	14	8	18
no. of corrections	5	4	1

## 4. Concluding remarks

Because of the need for unsupervised real-life data [35,36], the number of beyond-laboratory upper-limb prosthetic studies beyond the laboratory has been gradually increasing over the past 25 years [37]. These studies collected questionnaires, wear-time, grip count or video clips to investigate prosthetic interventions, device performances and user perspectives in home environments [13,38,39]. However, to fully understand and improve an advanced control algorithm for daily practice, more detailed control data and contextual information are necessary.

In this paper, we demonstrated a proof-of-principle study of applying IoT for prosthetic research beyond the laboratory. We demonstrated the system by a laboratory-based study, but the data collection, device monitoring and system updates were all carried out through Wi-Fi communication. Therefore, it is feasible to repeat the experiments in community settings without any system modification, as we proposed. The developed system saves the prosthetic control data to an online server and visualizes the data in real time through a web-based dashboard. User feedback forms and control panels on the dashboards allow the collection of contextual information and system reconfiguration over the air. Three able-bodied participants participated in the experiments. The experimenter could monitor the data in real time and reconfigure the device remotely.

The current version of this system saves timestamped myoelectric control signals, grasp commands and user intents, which will be sufficient for understanding how often and how well the controller is used in real-life settings. Future development may include expansion of the storage capacity to enable storing raw myoelectric and other sensor signals such as force and accelerometry. Transmitting raw signals in real time may require larger bandwidth and its necessity remains to be established. The raw myoelectric signals allow the experimenter to playback the muscle activity during at-home use and develop comprehensive datasets for further analysis, e.g. for machine learning-based decoding [40,41]. Meanwhile, transmitting the raw data will increase the resource consumption, in terms of bandwidth and storage space, but a large portion of them may not be used in scientific research due to the lack of contextual information [42]. Moreover, raw data transmission increases the power consumption, and it will significantly reduce the battery life of the device [43]. Considering these factors, further discussion may be required to determine whether it is worthwhile to transmit raw data or to convert myoelectric signals into features before transmission especially for high-density EMG settings [44–46]. If raw EMG signals are indispensable, they can be saved on the on-board SD card and uploaded to the server periodically. The proposed hardware is capable of the latter.

We respected data security and user privacy in our system design. Following the recommendations of [47], we adopted the default Thinger.io data security system design. First, each prosthesis controller had its ID and device credentials and needed to authenticate itself to the server before collecting or sending data. Besides, data were transmitted securely using the Transport Layer Security protocol. We further implemented user-level security such that the participants can see only their data and experimenters can only access projects for which they were responsible. In the send-home prosthetics research, in addition to cyber security, one should address users’ privacy concerns. As the perception of data sharing over the internet is different between people [48], we only collected the minimum data that were relevant to the study. Participants should have options to stop any kind of data sharing at any time, such as using the data-streaming buttons on the dashboards, so they can choose to share data over a specific period or allow us to for continuous monitoring [49]. The proposed hardware is capable of addressing all of these requirements.

With the support of IoT, the system can collect labelled real-life data during the pick and place experiment. These data include the style of the participant when doing specific muscle activities [50], so learning from them may yield a model that better fits the participant. We used the naive Bayes classifier as an example to demonstrate the optimization of the control model over the air. This is an example of how a simple machine-learning algorithm can promote data-driven clinical decision making, in the field of prosthetics. This approach helps in addressing ethical issues when using artificial intelligence in the healthcare domain [51]. Besides, it may increase the participants’ trust in the system, which can further enhance their experience and satisfaction with the intervention [52].

It is readily possible to adapt the proposed IoT system to incorporate alternative upper-limb prostheses and controllers. It is also possible to deploy the server on different platforms. Deploying a server on commercial IoT platforms simplifies the development processes by using the built-in functions for designing servers and the ready to use cloud infrastructure for connecting devices. They are feasible for pilot studies, but not all custom features are available in their libraries. As such, we were not able to introduce new modules to the server. For instance, we could not allow participants to add text comments to the motor commands because of the constraints on the widgets. A different approach from the demonstration is to self-host an open-source solution for data recording and user interfacing, e.g. [53]. The server has to be developed from sketch, but it is more flexible in the short run. However, the self-hosted server is a lot more challenging to scale up for long-term clinical trials.

Future works will focus on adapting the system on sent-home unsupervised studies, where participants with limb difference use our devices in their daily activities. The experimental design and the user interface will be co-created with a broad range of stakeholders so that users' needs and ethical considerations within user home environments can be better taken into account, as recommended by [54]. Besides, the real-time system configuration function will be deployed in the studies for not only the decision boundaries but also the gain and window length for each myoelectric channel. We envisage that this platform can be used to expedite the involvement of the clinicians in the research on prosthetics, especially when it comes to tuning the parameters of a prosthesis controller on the fly and with the involvement of users.

## Data Availability

The data are provided in electronic supplementary material [55]. The data that support the findings of this study are openly available in Edinburgh DataShare at https://doi.org/10.7488/ds/3260.
